# Interferon-inducible protein (IFI) 16 regulates Chikungunya and Zika virus infection in human skin fibroblasts

**DOI:** 10.17179/excli2019-1271

**Published:** 2019-06-27

**Authors:** Sineewanlaya Wichit, Rodolphe Hamel, Sakda Yainoy, Nuttamonpat Gumpangseth, Suchawadee Panich, Thanawat Phuadraksa, Phoonthawee Saetear, Arnaud Monteil, Ronald Morales Vargas, Dorothée Missé

**Affiliations:** 1 Department of Clinical Microbiology and Applied Technology, Faculty of Medical Technology, Mahidol University, Nakhon Pathom, 73170, Thailand; 2 Laboratoire MIVEGEC, IRD-CNRS-University of Montpellier, 34000, Montpellier, France; 3 Department of Microbiology and Immunology, Faculty of Tropical Medicine, Mahidol University, Bangkok, 10400, Thailand; 4 Department of Chemistry and Center of Excellence for Innovation in Chemistry, Faculty of Science, Mahidol University, Bangkok 10400, Thailand; 5 Plateforme de Vectorologie, BioCampus, Univ. Montpellier, CNRS, INSERM, 34094, Montpellier, France; 6 Department of Medical Entomology, Faculty of Tropical Medicine, Mahidol University, Bangkok, 10400, Thailand

**Keywords:** Chikungunya virus, Zika virus, IFI16, human skin fibroblasts, antiviral

## Abstract

Chikungunya virus (CHIKV), a re-emerging infectious arbovirus, causes Chikungunya fever that is characterized by fever, skin rash, joint pain, arthralgia and occasionally death. Despite it has been described for 66 years already, neither potential vaccine nor a specific drug is available yet. During CHIKV infection, interferon type I signaling pathway is stimulated and releases hundreds of interferon stimulated genes (ISGs). Our previous study reported that IFI16, a member of ISGs, is up-regulated during CHIKV virus infection and the suppression of the gene resulted in increased virus replication. Furthermore, our group also found that inflammasome activation can inhibit CHIKV infection in human foreskin cells (HFF1). Concomitantly, it has been reported that IFI16 activates the inflammasome to suppress virus infection. Therefore, we have hypothesized that IFI16 could be involved in CHIKV infection. In this study, we confirmed the expression level of IFI16 by Western blotting analysis and found that IFI16 was up-regulated following CHIKV infection in both HFF1 and human embryonic kidney cells. We next investigated its antiviral activity and found that forced expression of IFI16 completely restricted CHIKV infection while endogenous silencing of the gene markedly increased virus replication. Furthermore, we have discovered that IFI16 inhibited CHIKV replication, at least, in cell-to-cell transmission as well as the diffusion step. Interestingly, IFI16 also exerted its antiviral activity against Zika virus (ZIKV) infection, the global threat re-emerging virus can cause microcephaly in humans. Taken together, this study provides the first evidence of an antivirus activity of IFI16 during *in vitro* arbovirus infection, thus expanding its antiviral spectrum that paves the way to further development of antiviral drugs and vaccines.

## Introduction

Chikungunya virus (CHIKV) is an emerging infectious virus belonging to the family *Togaviridae*, genus alphavirus. CHIKV was named Makonde “kungunyala” which means “that which bends up”, referring to the posture of patients suffering from severe joint pain during CHIKV infection, after it was first reported in Makonde plateau (Southern Tanzania) in 1952 (Robinson, 1955[22]). The virus can be transmitted by the bite of mosquito vectors including *Aedes (Ae.) *aegypti and *Ae. Albopictus *which occurrs initially in human skin. Due to the huge expansion of mosquito vectors the prevalence of CHIKV now spreads approximate 60 countries throughout tropical and sub-tropical areas in the world (Wahid et al., 2017[25])*.* CHIKV infection in humans causes Chikungunya fever (CHIKF) which is a febrile associated with high fever, rash, headache and can be a chronic condition that lasts for years (Hamer, 2019[6]). The increasing emergence of CHIKV urgently needs a specific treatment to minimize the symptoms and morbidity. Until now, owing to the absence of vaccine against CHIKV infection or its treatment, extensive research efforts have been directed towards the development of suitable antiviral strategies. 

During CHIKV infections, type I interferons (IFNs) are known to be produced and to initiate the production of pro-inflammatory cytokines. Interferon stimulated genes (ISGs) against the virus infection are triggered (Lum and Ng, 2015[15]). Understandably, our previous study reported that inhibition of IFN type I signaling pathway leads to the enhancement of CHIKV replication (Wichit et al., 2017[26]). Interestingly, in a similar study, interferon-inducible protein 16 (IFI16) expression level markedly decreases while an increase of virus was observed.

IFI16 is a member of the PYHIN protein family (also known as p200 or HIN-200 proteins) composed of a Pyrin domain, a protein-protein interaction site, and two HIN domains (HinA and HinB), a non-specific dsDNA binding domain (Unterholzner et al., 2010[24]; Jin et al., 2012[9]). Previous studies have reported that IFI16 can activate innate antiviral immune responses by acting as a nuclear pathogen sensor to induce the inflammasome (Kerur et al., 2011[11]) or trigger the STING-TBK1-IRF3 of IFN I signaling pathway (Orzalli et al., 2012[20], 2015[18]; Li et al., 2013[13]; Thompson et al., 2014[23]) in order to inhibit Herpes simplex virus (HSV), Human cytomegalovirus (HCMV) as well as Sendai virus (SeV). Moreover, IFI16 was also described as a viral restriction vector against ICP0-null HSV-1 and HCMV in both fibroblasts and keratinocytes (Gariano et al., 2012[4]; Orzalli et al., 2013[19]; Johnson et al., 2018[10]; Merkl et al., 2018[17]) and human papillomavirus 18 (HPV18) in U2OS cells (Lo Cigno et al., 2015[14]). Recently, Merkl and Knipe explained the IFI16 restriction role. As a matter of fact, they reported that IFI16 can recruit the other restriction factors such as the promyelocytic leukemia (PML), Sp100 and ATRX to form nuclear filamentous structures named “restrictosome” which signals in *cis* and *trans* to suppress the progeny viral DNA (Merkl and Knipe, 2019[16]). Although, there are many reports describing an antiviral function of IFI16, there is no evidence of its role in CHIKV infection which is the global threat arbovirus.

As, IFI16 is involved in the inflammasome activation via the induction of caspase-1 and IL-1β, this results in the regulation of herpesvirus KSHV (Merkl et al., 2018[17]). Concomitantly, our previous study has reported the responsiveness of IFI16 expression level during CHIKV infection (Wichit et al., 2016[27]). Moreover, we described the antiviral activity involvement of the inflammasome in the main route of transmission via human foreskin cells (Ekchariyawat et al., 2015[3]). Thus, we hypothesized that IFI16 should be implicated on CHIKV replication. In this study, we investigated the antiviral activity of IFI16 in CHIKV replication on HFF1. We found that intrinsic IFI16 expression level was up-regulated during CHIKV infection in human skin cells and epithelial from human embryonic kidney cells. Interestingly, overexpression of IFI16 suppressed CHIKV infection while its knockdown clearly enhanced the viral replication in human skin fibroblasts.

## Materials and Methods

### Cells and virus

C6/36 *Ae. albopictus* cells, used for propagation of the CHIKV strains, were grown at 28 °C in Dulbecco's modified Eagle's medium (DMEM; Invitrogen) supplemented with 10 % fetal calf serum (FCS; Lonza) as previously described (Wichit et al., 2017[28]). The HFF1 human skin fibroblast cell line, Vero cells (African green monkey cell line) and HEK293T (Human embryonic kidney cell line) were maintained in DMEM supplemented with 15 %, 5 % and 10 % FCS, respectively (Wichit et al., 2017[28]). All cell lines were purchased from ATCC.

The low-passage-number of the CHIKV LR2006_OPY1 strain (Wichit et al., 2017[28]), was isolated from a viremic patient in La Réunion Island in 2006. The clinical isolate PF-25013-18 of ZIKV has been previously described (Hamel et al., 2015[5]). All viruses were grown in C6/36 cells at 28 °C and kept at -80 °C until used.

### Antibodies, reagents and plasmid

Rabbit monoclonal anti-IFI16 antibody was purchased from Santa Cruz Biotechnology, mouse monoclonal anti-β-actin antibody, anti-rabbit IgG HRP conjugated antibody, anti-Mouse IgG HRP conjugated antibody, thiazolyl blue tetrazolium bromide (MTT) were purchased from Sigma-Aldrich (St Louis, MO). The IFI16-FL-encoding cDNA cloned in the pcDNA3-FLAG expression plasmid was obtained from Addgene (#35064). Lipofectamine 2000 was purchased from Invitrogen.

### Viability assay

Cell viability was determined using a MTT-based assay. Briefly, cells were transfected with the IFI16 plasmid and incubated at 37 °C, 5 % CO_2_ for 24 hours (h), washed by phosphate-buffered saline (PBS) and then incubated with 100 µL MTT. After 2 h, MTT was removed and 50 µL of DMSO was added to each well and mixed thoroughly. The mixture was incubated at 37 °C for 10 min and cellular viability was determined by measuring the absorbance value at 570 nm.

### Viral infection

Human fibroblasts were seeded in six-well plates and grown to 70-80 % confluence. The cultures were rinsed twice with PBS and the cells were incubated with CHIKV or ZIKV at the desired multiple of infection (MOI) for 2 h at 37 °C while gently agitating the plates. Then, the inoculum was removed and the cells were washed three times with PBS. DMEM supplemented with 15 % FCS was added to each well and the plates were incubated at 37 °C and 5 % CO_2_, for the duration of the experiment. Cells serving as a negative control were incubated with culture supernatant from uninfected C6/36 cells.

### Transfection

Human skin fibroblasts cells were seeded in six-well plates. After a 24 h incubation at 37 °C with 5 % CO_2_, plasmids were transfected with Lipofectamine 2000 according to the manufacturer's protocol. The transgenes expression were evaluated by Western blot analysis and RT-qPCR.

### Plaque assay

Vero cells, grown to 70-80 % confluence, were incubated with four separate, ten-fold, dilutions of viral supernatant in DMEM at 37 °C for 2 h. Then, a mix of nutriment solution with agar (Lonza) was added and the cells were maintained at 37 °C for 5 days. For plaque counting, the cells were incubated with 3.7 % formaldehyde and 0.5 % Crystal violet in 20 % ethanol.

### Western blotting analysis

Cells were lysed on ice in RIPA buffer (150 mM NaCl, 5 mM β-mercaptoethanol, 1 % NP-40, 0.1 % sodium dodecyl sulfate, 50 mM Tris-HCl, pH 8) supplemented with protease inhibitor cocktail solution (Sigma-Aldrich). The protein concentration was determined by bicinchoninic acid (BCA) assay (Thermo Scientific). Equal amounts of proteins were mixed with *Laemmli* sample loading buffer, heated for 5 min at 100 °C, subjected to SDS-PAGE and electrotransferred onto a nitrocellulose membrane. The membrane was blocked with 0.05 % Tween 20 in PBS (PBST) containing 5 % skim milk for 1 h at room temperature (RT), then incubated overnight at 4 °C with the desired primary antibody. Membranes were then washed three times with PBST, and subsequently incubated for 1 h at RT with horseradish peroxidase-coupled secondary antibodies (Cell Signaling) in PBST. The membrane was washed three times, and proteins were detected by chemiluminescence using a SuperSignal West Pico chemiluminescent substrate kit (Thermo Scientific). The membrane was then stripped and re-probed with an anti-β-actin antibody to ensure that equivalent levels of protein were loaded in each lane.

### Gene expression by real-time PCR analysis

Total RNA was extracted from fibroblasts using Tri reagent (Sigma-Aldrich) following the manufacturer's protocol. cDNA was synthesized using 0.5 μg of total RNA and the MMLV reverse transcription Kit (Promega). The expressions of genes were quantified by RT-qPCR using the Roche Light Cycler LC480 device and specific primers (Table 1(Tab. 1)). All data are presented as mean ± SD. A comparison of real-time PCR values was made with the student *T*-test. For all experiments, statistical significance was accepted at p < 0.05.

### Viral RNA quantification by real-time RT-PCR

Total RNA was extracted from human fibroblasts by using Tri reagent (Sigma-Aldrich). The RNA pellet was resuspended in 25 μl of RNase-free distilled water and stored at -80 °C. The RNA was then used for reverse transcription using MMLV reverse transcription Kit (Promega) according to the manufacturer's instructions. The reaction was carried out using 1 μg total RNA as template for the normalization of viral RNA to the amount of total RNA. The MaximaTM Probe/ROX qPCR Master Mix (2x) (Thermo Scientific) was used in qPCR experiments. Each reaction of 25 μL contained 400 nM of each primer, 200 nM of specific probe and 1x Maxima Probe/ROX qPCR Master Mix. 

Primers and probe sequences are listed in Table 1(Tab. 1). The amplification conditions were 95 °C for 10 min followed by 45 amplification cycles of 95 °C for 15 s, 60 °C for 20 s and 72 °C for 30 s. The reactions were performed in an Applied Biosystem 7300 system. Real-time data were analyzed using the SDS software (Thermo Fisher Scientific). Viral RNA was quantified by comparing the sample's threshold cycle (Ct) values with each virus RNA standard curve which was obtained as previously described (Wichit et al., 2017[28]).

### Data analysis and statistical methods

All data are presented as means ± SD. A comparison of qPCR values was made with the student *T*-test. For all experiments, statistical significance was accepted at p < 0.05.

## Results

### Chikungunya virus up-regulates IFI16 expression in multiple cell lines

**Figure 1:**
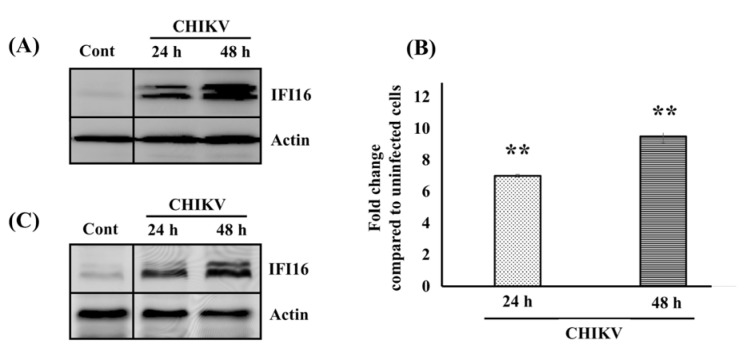
CHIKV infection enhances IFI16 expression level in HFF1 and HEK293T. (A) HFF1 cells were either uninfected (Cont) or infected with CHIKV for 24 and 48 hpi. Then, infected cells were lysed for detection of IFI16 and Actin by Western blotting analysis. HEK293T cells were either uninfected (Cont) or infected with CHIKV for 24 and 48 hpi. (B) Infected cells were harvested either by Trizol for IFI16 mRNA quantification using qRT-PCR or (C) by lysis buffer for IFI16 and Actin detection using Western blotting analysis.

As we previously reported, the expression of endogenous IFI16 is up-regulated both at the mRNA level and protein level in human skin fibroblasts (HFF1) as assayed by qPCR Array and SILAC analysis, respectively (Wichit et al., 2017[26], 2019[29]). In the present study, we confirmed these data. As a matter of fact, Western blotting analysis against IFI16 was performed and we found an increase in the amount of IFI16 in CHIKV-infected human fibroblasts in a time-dependent manner (Figure 1A(Fig. 1)). In addition, we next extended our finding by detecting the expression of endogenous IFI16 in the human embryonic kidney cell line (HEK293T), a CHIKV permissive cell line (Krejbich-Trotot et al., 2011[12]). To address this point, HEK293T cells were infected by CHIKV. The IFI16 expression level was measured at 24 and 48 hours after infection (hpi.). As observed with HFF1 cells, the mRNA as well as protein levels of IFI16 were up-regulated in a time-dependent manner in HEK293T cells (Figure 1B and 1C(Fig. 1)). The results indicated that intrinsic IFI16 responded to CHIKV infection indicating that it could play an important role in virus infection. In order to investigate effects of IFI16 on CHIKV and ZIKV infection, IFI16-overexpressed and silenced human skin fibroblasts were used as a model.

### Expression of IFI16 restricts Chikungunya virus infection

**Figure 2:**
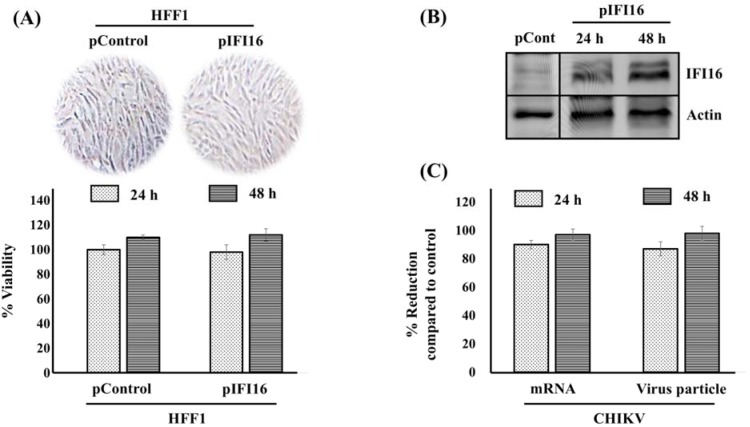
Overexpression of IFI16 in human skin fibroblasts inhibits CHIKV infection. HFF1 cells were transfected by either pControl or pIFI16 using lipofectamine 2000. After 24 and 48 h, transfected cells were (A) visually inspected to detect putative morphological changes and cell death, then, % viability was measured using a MTT assay. (B) IFI16 expression level was confirmed by Western blotting analysis. (C) IFI16 overexpressed cells were challenged with CHIKV for 24 and 48 h. Virus mRNA and virus particles were then quantified by qRT-PCR and plaque assay, repectively. Results are presented as mean ± SD from three independent experiments. % Reduction was calculated using the formular [1-(R/C)]*100 where C and R designate experimental values (RNA copy numbers or plaque numbers) in the presence of pCtrl and pIFI16, respectively.

Our previous study demonstrated the immune sensor, AIM2, reduces CHIKV replication by the induction of the inflammasome in HFF1 cells (Ekchariyawat et al., 2015[3]). Similarly, IFI16, is an immune sensor that belongs to the HIN200 family similarly to AIM2. Both proteins were gathered in a new class of pattern recognition receptors (PRRs) called AIM2-like receptors (ALRs) that can detect microbial DNA (Connolly and Bowie, 2014[2]). Besides, several studies reported that IFI16 can inhibit various types of DNA viruses (Broz and Monack, 2013[1]). However, it is not known whether this inhibition may occur for RNA virus. Therefore, we examined the effects of IFI16 on CHIKV replication in HFF1 cells. IFI16-overexpressing HFF1 cells were generated at the optimum concentration without any cytotoxicity (Figure 2A(Fig. 2)). The protein expression level of IFI16 was confirmed by Western blot analysis (Figure 2B(Fig. 2)). Cells were subsequently infected with CHIKV at a MOI of 1 and the extent of virus replication was analysed by quantifying the mRNA level and the number of virus particles. We found that both the viral RNA level and the virion production of the virus were almost totally abrogated in infected fibroblasts that overexpressed IFI16 compared to the control condition after 24 and 48 hpi. (Figure 2C(Fig. 2)). Together, these results revealed that forced expression of IFI16 could inhibit CHIKV replication, at least, in human skin cells.

### Depletion of IFI16 enhances Chikungunya virus infection

**Figure 3:**
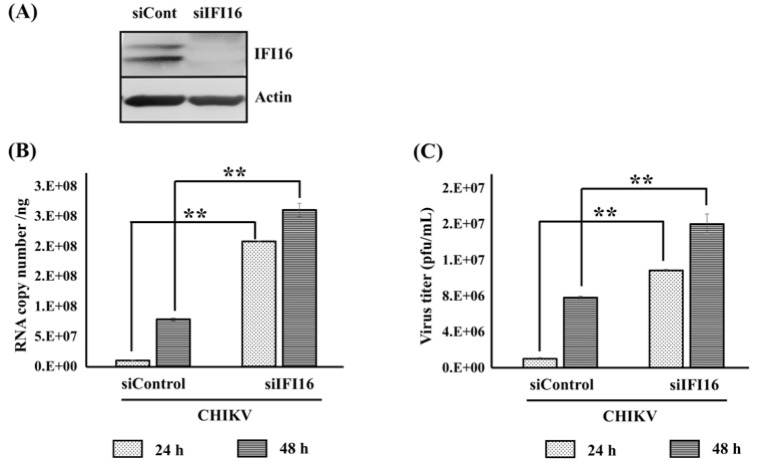
Knockdown of IFI16 enhances CHIKV replication in human skin fibroblasts. HFF1 cells were transfected with either siControl or siRNA targeting IFI16 (siIFI16) using lipofectamine 2000. (A) The knockdown was confirmed by Western blotting analysis 24 h post transfection. (B) Cells were challenged with CHIKV for 24 and 48 h. The viral replication was quantified at the mRNA level by RT-qPCR and (C) number of virus particles were determined by plaque assay. The results are represented by mean ± SD of three independent experiments. **, p < 0.01, as compared to siControl transfected cells.

In order to confirm the antiviral role of IFI16, we next investigated whether depletion of IFI16 had any impact on CHIKV replication. The expression of endogenous IFI16 was knocked down using a specific siRNA. The knockdown was confirmed by Western blotting analysis (Figure 3A(Fig. 3)). Cells were then infected by CHIKV for 24 and 48 h before quantification of the virus replication by qRT-PCR and plaque assay. As expected, our results showed a significant increase of CHIKV viral mRNA and viral particle compared to control cells (Figure 3B and 3C(Fig. 3)). These data perfectly support an antiviral role of IFI16 in CHIKV infection by indicating that the expression level of IFI16 modulates viral replication.

### IFI16 suppressed the early stages, cell-to-cell transmission and diffusion of the Chikungunya virus infection

**Figure 4:**
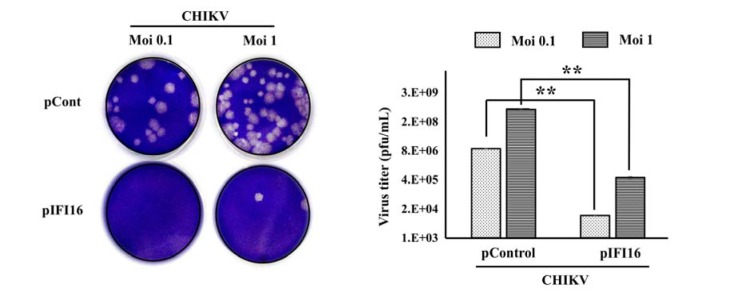
IFI16 restricts CHIKV infection in an early stage of infection. Vero cells were transfected by pIFI16 for 24 h before being infected by CHIKV at MOIs of 0.1 and 1. Infected cells were fixed, stained and counted in order to quantify the number of virus particles. The results were represented as mean ± SD of three independent experiments. **, p < 0.01, as compared to pControl transfected cells.

To further determine which step of the viral replication cycle is impacted by IFI16, cell-to-cell transmission and diffusion experiments were carried out. IFI16-overexpressing vero cells or control cells were infected with CHIKV. Cells were fixed and stained by crystal violet to visualize the plaque formation. After 4 days, expression of IFI16 in vero cells strongly suppressed the cytopathic effect (CPE) and plaque formation of virus infection (Figure 4(Fig. 4)). These results suggested that IFI16 has an impact on the CHIKV replication cycle, at least, in the early stage of the virus replication cycle.

### IFI16 also exerts its antiviral activity against Zika virus infection

**Figure 5:**
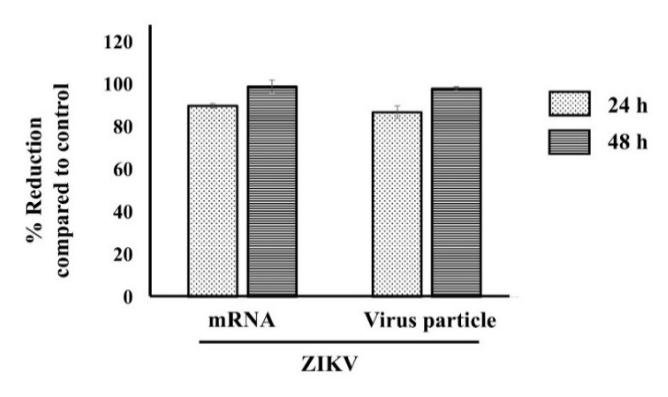
IFI16 exerts its antiviral activity to ZIKV infection in HFF1 cells. IFI16 overexpressing cells were challenged with ZIKV for 24 and 48 h. Virus mRNA and virus particle were then quantified by RT-qPCR and plaque assay, respectively. The results were represented mean ± SD of three independent experiments. % Reduction was calculated using the formular [1-(R/C)]*100 where C and R designate experimental values (RNA copy numbers or plaque numbers) in the presence of pCtrl and pIFI16, respectively.

Since the beginning of the 21st century, the emergence of CHIKV and ZIKV has taken place globally and is a major worldwide public health problem (Huang et al., 2019[7]). In order to investigate whether the antiviral effects of IFI16 are limited only to CHIKV or may be extended to other viruses, we extended our experiment to determine a putative antiviral effect on ZIKV, a member of the genus *Flavivirus*, in human skin fibroblasts. Similarly to what was observed for CHIKV, IFI16 strongly inhibited the replication of ZIKV. RNA replication and virion production of virus were significantly inhibited and approached 100 %, without any deleterious effects on cell viability (Figure 5(Fig. 5)). Thus, our findings for a role of IFI16 in CHIKV are extendable to ZIKV.

## Discussion

CHIKV belongs to the alphavirus family transmitted by the *Aedes spp. *by the bite of human skin, a major cell type for viral entry. During infection, we have found that CHIKV-infected human fibroblasts expressed a significantly increased level of various type I IFN-responsive genes including the Interferon-γ-inducible protein 16 (IFI16) (Wichit et al., 2017[26]). In the same study, CHIKV infection in the presence of mosquito saliva markedly decreased IFI16 expression level resulting in the significant increase of viral transcripts and infectious viral particles. IFI16 was first reported as a nuclear innate DNA sensor that can activate the inflammasome ultimately helping to fight infection (Kerur et al., 2011[11]). Correlated with our finding, it was shown that the inflammasome signaling pathways exert antiviral effects against CHIKV *via* the activation of IL-1β, maturation of caspase-1 and expression of the inflammasome sensor AIM2 (Ekchariyawat et al., 2015[3]). These findings highlight a crucial role of IFI16 on CHIKV infection. Several groups proposed the antiviral activity of IFI16 against DNA virus infections such as adenovirus-based vector AdVIFI16, EBV, HCMV, HSV-1, HPV and retrovirus such as HIV-1 (Gariano et al., 2012[4]; Jakobsen et al., 2013[8]; Lo Cigno et al., 2015[14]; Pisano et al., 2017[21]; Johnson et al., 2018[10]). We extend herein these findings about the antiviral activity of IFI16 to arbovirus infection.

First, we confirmed our previous work which reported an up-regulation of IFI16 mRNA level after CHIKV infection (Wichit et al., 2017[26], 2019[29]). We showed an up-regulation of IFI16 protein after CHIKV infection in both human cutaneous fibroblast cell line (HFF1) and human embryonic kidney cells (HEK293T), which extended our previous findings. Indeed, it has been shown that IFI16 expression could be induced by the vaccinia virus which is a DNA virus (Unterholzner et al., 2010[24]). This similar finding may be explained by the ability of IFI16 to act as an immune sensor for virus infection (Kerur et al., 2011[11]). We are the first group to report the intrinsic activation of IFI16 by CHIKV. Whether this up-regulation of IFI16 in infected cells confers protection to hitherto uninfected cells must be clarified in further experiments. Secondly, the overexpression of IFI16 completely restricted CHIKV infection, and at least, cell-to-cell transmission and diffusion. Therefore, it is likely that IFI16 up-regulation could protect uninfected bystander cells. In addition, our results appeared extendable to other arbovirus as we found IFI16 also exerted its antiviral activity against ZIKV infection. These findings are strongly supported by the defection of type I IFN production which normally increases during CHIKV infection to protect the cells. This was explained by the fact that IFI16 knockdown in cells may result in less recruitment of RNA polymerase II (Pol II) to the IFN-α promoter leading to a defect in IFN-α production and, result in reduced basal levels of ISGs (Thompson et al., 2014[23]). Recently, the reduction of RNA Pol II recruitment by IFI16 was proposed. IFI16 forms nuclear filamentous structures called a “restrictosome” that appear to signal to progeny viral genomes throughout the infected cell nucleus to cause their epigenetic silencing and reduce expression of the viral genes (Merkl and Knipe, 2019[16]).

## Conclusion

In conclusion, we provide the first report showing the crucial role of IFI16 antiviral activity against members of arbovirus including CHIKV and ZIKV. Many mechanisms of IFI16-mediated virus suppression have been reported including an action as a restriction factor, as an interference of innate immune responses and as a filamentous nuclear assembly, however, the antiviral mechanisms involved in arbovirus infection remain unclear and require to be investigated in further studies. Although, more work is required, our study provides the novel potentially new host factor that can inhibit arbovirus infection, at least, in the early stages of infection. Thus our work paves the way to the development of novel antiviral strategies to fight arbovirus infections in humans.

## Acknowledgements

This work was funded by Thailand Research Fund (TRF) (grant no. MRG6280009).

## Conflict of interest

The authors declare that they have no conflict of interest.

## Figures and Tables

**Table 1:**
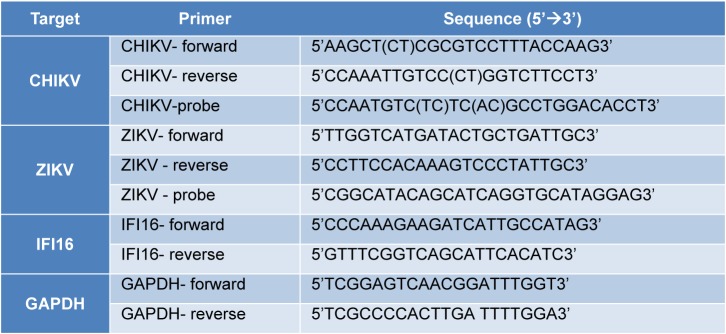
Primers and probes sequences used in this study
